# Grapefruit seed extract effectively inhibits the *Candida albicans* biofilms development on polymethyl methacrylate denture-base resin

**DOI:** 10.1371/journal.pone.0217496

**Published:** 2019-05-28

**Authors:** Chiaki Tsutsumi-Arai, Kensuke Takakusaki, Yuki Arai, Chika Terada-Ito, Yusuke Takebe, Takahiro Imamura, Shinji Ide, Seiko Tatehara, Reiko Tokuyama-Toda, Noriyuki Wakabayashi, Kazuhito Satomura

**Affiliations:** 1 Department of Oral Medicine and Stomatology, Tsurumi University School of Dental Medicine, Tsurumi, Tsurumi-ku, Yokohama, Kanagawa, Japan; 2 Department of Removable Partial Prosthodontics, Graduate School, Tokyo Medical and Dental University (TMDU), Yushima, Bunkyo-ku, Tokyo, Japan; Center for Cancer Research, UNITED STATES

## Abstract

This study aimed to investigate the cleansing effects of grapefruit seed extract (GSE) on biofilms of *Candida albicans* (*C*. *albicans*) formed on denture-base resin and the influence of GSE on the mechanical and surface characteristics of the resin. GSE solution diluted with distilled water to 0.1% (0.1% GSE) and 1% (1% GSE) and solutions with Polident® denture cleansing tablet dissolved in distilled water (Polident) or in 0.1% GSE solution (0.1% G+P) were prepared as cleansing solutions. Discs of acrylic resin were prepared, and the biofilm of *C*. *albicans* was formed on the discs. The discs with the biofilm were treated with each solution for 5 min at 25°C. After the treatment, the biofilm on the discs was analyzed using a colony forming unit (CFU) assay, fluorescence microscopy, and scanning electron microscopy (SEM). In order to assess the persistent cleansing effect, the discs treated with each solution for 5 min were aerobically incubated in Yeast Nitrogen Base medium for another 24 h. After incubation, the persistent effect was assessed by CFU assay. Some specimens of acrylic resin were immersed in each solution for 7 days, and changes in surface roughness (Ra), Vickers hardness (VH), flexural strength (FS), and flexural modulus (FM) were evaluated. As a result, the treatment with 1% GSE for 5 min almost completely eliminated the biofilm formed on the resin; whereas, the treatment with 0.1% GSE, Polident, and 0.1% G+P for 5 min showed a statistically significant inhibitory effect on biofilms. In addition, 0.1% GSE and 0.1% G+P exerted a persistent inhibitory effect on biofilms. Fluorescence microscopy indicated that Polident mainly induced the death of yeast, while the cleansing solutions containing at least 0.1% GSE induced the death of hyphae as well as yeast. SEM also revealed that Polident caused wrinkles, shrinkage, and some deep craters predominantly on the cell surfaces of yeast, while the solutions containing at least 0.1% GSE induced wrinkles, shrinkage, and some damage on cell surfaces of not only yeasts but also hyphae. No significant changes in Ra, VH, FS, or FM were observed after immersion in any of the solutions. Taken together, GSE solution is capable of cleansing *C*. *albicans* biofilms on denture-base resin and has a persistent inhibitory effect on biofilm development, without any deteriorations of resin surface.

## Introduction

*Candida albicans* (*C*. *albicans*), which is frequently isolated from denture plaque, is the main microorganism involved in the pathogenesis of denture stomatitis (DS) [1]. As *C*. *albicans* has a high affinity to acrylic resin denture-base material, it adheres easily to and forms a dense biofilm on denture surfaces [2, 3]. Therefore, one of the most effective methods of treating and preventing DS is the removal of this fungus from the denture surfaces by efficient cleansing.

The most commonly recommended method for cleaning dentures is a combination of brushing and immersion in a chemical cleanser [4]. However, it is difficult for patients with limited motor capacity to sufficiently clean the dentures with brushing [5, 6]; therefore, a large amount of plaque can remain on denture surfaces. In these cases, a long-term immersion in chemical cleansers would be necessary for removal of the residual plaque. Although chemical cleansers can reduce microbial viability and biomass volume on denture surfaces, viable microorganisms remain, which may cause DS [7, 8]. In addition, many studies have shown that long-term immersion in some cleansers causes degradation of the physical/mechanical properties and color of denture-base resin and metal alloy [9, 10]. For these reasons, a new cleanser that can remove denture plaque more effectively and in a shorter time than conventional cleansers should be developed.

Grapefruit seed extract (GSE), which contains large quantities of polyphenolic compounds, is a natural extract derived from plants [11, 12]. Flavonoids are a class of polyphenols, and they account for approximately 80% of polyphenolic compounds in GSE [11]. It has been reported that flavonoids have an inhibitory effect on gram-positive and gram-negative bacteria and some *Candida* species by various mechanisms [13–17]. Indeed, GSE is reported to have powerful antibacterial and antifungal properties [12, 18, 19], and it has been studied extensively as a natural preservative of food [20–23]. These studies suggest that GSE has the potential to cleanse *C*. *albicans* attached to denture. However, no studies have investigated the effect of GSE for cleansing *C*. *albicans* biofilm formed on the denture-base resin.

This study aimed to investigate the cleansing effect of GSE on *C*. *albicans* biofilms formed on denture-base resin and the effects of GSE on the mechanical and surface characteristics of the resin.

## Materials and methods

### Preparation of cleansing solutions

An original (100%) solution of GSE (Will Tool, Biochemical Technical Laboratory Co., Ltd., Niigata, Japan) was supplied by EID Inc. (Tokyo, Japan). GSE was diluted to 0.1% and 1% with distilled water, and the solutions were referred to as “0.1% GSE” and “1% GSE,” respectively. A Polident denture cleansing tablet (GlaxoSmithKline, Weybridge, UK) was dissolved in 150 mL distilled water or 150 mL 0.1% GSE, and the solutions were referred to as “Polident” and “0.1% G+P,” respectively.

### Specimen preparation and biofilm production

A set of 250 polymethyl methacrylate (PMMA) denture-base resin discs (10 mm in diameter and 2 mm in thickness) (ACRON, GC, Tokyo, Japan) was prepared using the method reported in our previous study [24], and the upper and lower surfaces of each disc were polished with abrasive paper (320 grit) under dry conditions. The surface roughness (Ra) of all discs was determined by a profilometer (Surfcom Flex, Tokyo Seimitsu, Tokyo, Japan), where the mean value calculated from two measurements was 1.12 ± 0.15 μm. All discs were sterilized with ethylene oxide gas (EOG), stored in a sterilization chamber at 40°C for 24 h to remove residual EOG, then immediately used for the following investigations.

Each disc was placed in one of the wells of a 24-well plate with artificial saliva [500 μL; 1.25 mM Ca(NO_3_)_2_∙4H_2_O, 0.90 mM KH_2_PO_4_, 129.91 mM KCl, 59.93 mM Tris buffer, and 2.2 g/L porcine gastric mucin; pH 7.4] [24, 25]. The plates were incubated for 60 min (37°C on a shaker at 75 rpm) and washed twice with 1 mL of phosphate-buffered saline (PBS; pH 7.2). *C*. *albicans* (ATCC18804) was grown in Tryptic Soy Broth supplemented with 5% dextrose (TSBD; Becton, Dickinson and Company, NJ, the USA) on a shaker at 75 rpm for 5 h at 30°C. The yeast-like cells were standardized at 10^6^ cells/mL in TSBD medium using a counting chamber (One cell counter, Biochemical Science, Tokyo, Japan). The *Candida* cell suspension (1 mL) was added to each disc-containing well. All well plates were aerobically maintained for 1.5 h at 37°C during the cell adhesion phase [25], after which all specimens were washed twice with 1 mL of PBS to remove the non-adhered cells. Then, the Yeast Nitrogen Base (YNB) medium (1 mL) was added to each well, and all the plates were aerobically incubated for 72 h at 37°C with changing the medium every 24 h. After the incubation, all specimens were washed twice with PBS.

### Biofilm analyses

The discs with a biofilm formed by *C*. *albicans* were treated with 1 mL of each cleansing solution or distilled water (as a control) for 5 min. The treatment time of the discs to each cleansing solution was determined based on the time (5 min) stated on the pack instructions of Polident denture cleansing tablet. After the treatment, the cleansing effect of each solution was assessed by colony forming unit (CFU) assay, and the discs treated with each solution were examined by fluorescence microscopy and scanning electron microscopy (SEM). In order to assess the persistent cleansing effect, the discs treated with each solution for 5 min were aerobically incubated again in one of the wells of a 24-well plate with the YNB medium (1 mL) for 24 h at 37°C. After the incubation, the persistent effect of each solution was assessed by CFU assay, and colony counts were compared before and after the 24-h incubation.

### CFU assay

The discs treated with each solution for 5 min were placed in one of the wells of a 24-well plate, and 1 mL of PBS was added to each well. The biofilm was scraped from each disc using a cell scraper (Iwaki Co., Tokyo, Japan), and the dissociated cells were suspended by pipetting up and down [24, 25]. The fungal suspensions were serially diluted with PBS, and 100-μL aliquot of each suspension was inoculated on Sabouraud glucose agar plates. After aerobic incubation for 48 h at 37°C, the CFUs were counted. Regarding the discs incubated for 24 h after the treatment, CFU assay was also conducted in the same way. Twenty discs from each group were used for this assay.

### Fluorescence microscopy

The discs treated with each solution for 5 min were placed in the wells of a 24-well plate, and 500 μL of physiological saline (Otsuka Pharmaceutical Co., Tokyo, Japan) was added to each well. Thereafter, the biofilms on the discs were stained using the Live/Dead FungaLight yeast viability kit, which was composed of SYTO-9 and propidium iodide (PI). In brief, 1 μL of SYTO9 and 1 μL of PI were added into each well, and the discs were incubated for 20 min in the dark at 30°C. The stained discs were observed using a fluorescence microscope (BZ-X710; Keyence, Osaka, Japan). Five discs from each group were used for this assay.

### Scanning electron microscopy (SEM)

The discs treated with each solution for 5 min were fixed overnight with 2.5% glutaraldehyde at 4°C. After fixation, each disc was dehydrated with graded concentrations of ethanol (50%, 60%, 70%, 80%, 90%, and absolute ethanol), transferred into liquidized t-butyl alcohol, and maintained in a freezer (-20°C) until the butyl alcohol froze. Thereafter, the specimens were transferred into a freeze-drying apparatus (ID-2; Eiko Engineering, Tokyo, Japan), and the frozen t-butyl alcohol was completely sublimated. Then, the specimens were mounted on aluminum stubs, sputter-coated with gold in an ion sputter coater (SC-701AT, Sanyu Denshi, Tokyo, Japan), and assessed using SEM (JCM-6000 NeoScope, Jeol Ltd., Tokyo, Japan). The morphological changes of the yeast and hyphal forms treated with each solution were observed. Five discs from each group were used for this assay.

### Characterization of denture-base resin after treatment with each cleansing solution

Fifty rectangular parallelepiped specimens (64×10×3.3 mm) were prepared using ACRON. The specimens were polished with 1000-grit abrasive paper under running water using a polishing device (ML-150P; MARUTO INSTRUMENT CO., LTD., Tokyo, Japan.). The specimens were immersed in each solution for 7 days (n = 10 in each group). Each solution was freshly prepared and changed every day. Given that the denture was immersed in these solutions for 5 min a day, the immersion time of 7 days was equal to about 5 and a half years. After the immersion, all specimens were stored at 25°C for one week, and the Ra of five specimens which were selected randomly was measured. Five specimens of each group were used for the Vickers hardness (VH) test, which was performed with a diamond hardness indenter (AVK-AII, Akashi Seisakusho, Tokyo, Japan) under a 300 g load for 15 s. Five indentations were made at different points for each specimen, and the means of individual specimens were calculated. Then, the remaining five specimens of each group were stored in deionized water for 50 h at 37°C. A three-point bending test was performed according to ISO 1567 with a universal testing machine (AG-5kNX plus, Shimadzu, Kyoto, Japan) at a crosshead speed of 1 mm/min to investigate flexural strength (FS) and modulus (FM). FM was calculated from the linear portion of the load-time curve up to the proportional limit obtained by the bending test.

### Statistical analysis

In order to confirm significant differences among the experimental groups, CFU assay results were analyzed using the Kruskal–Wallis test, and significant differences among the experimental groups were confirmed using the Mann–Whitney U test and the Bonferroni correction. Results for Ra, VH, FS, and FM of each specimen were subjected to one-way analysis of variance, followed by Tukey's significant difference multiple comparison test. The significance level was set at 0.05. All analyses were performed using SPSS ver. 24.0 for Windows (IBM, NY, USA).

## Results

**Fig 1:**
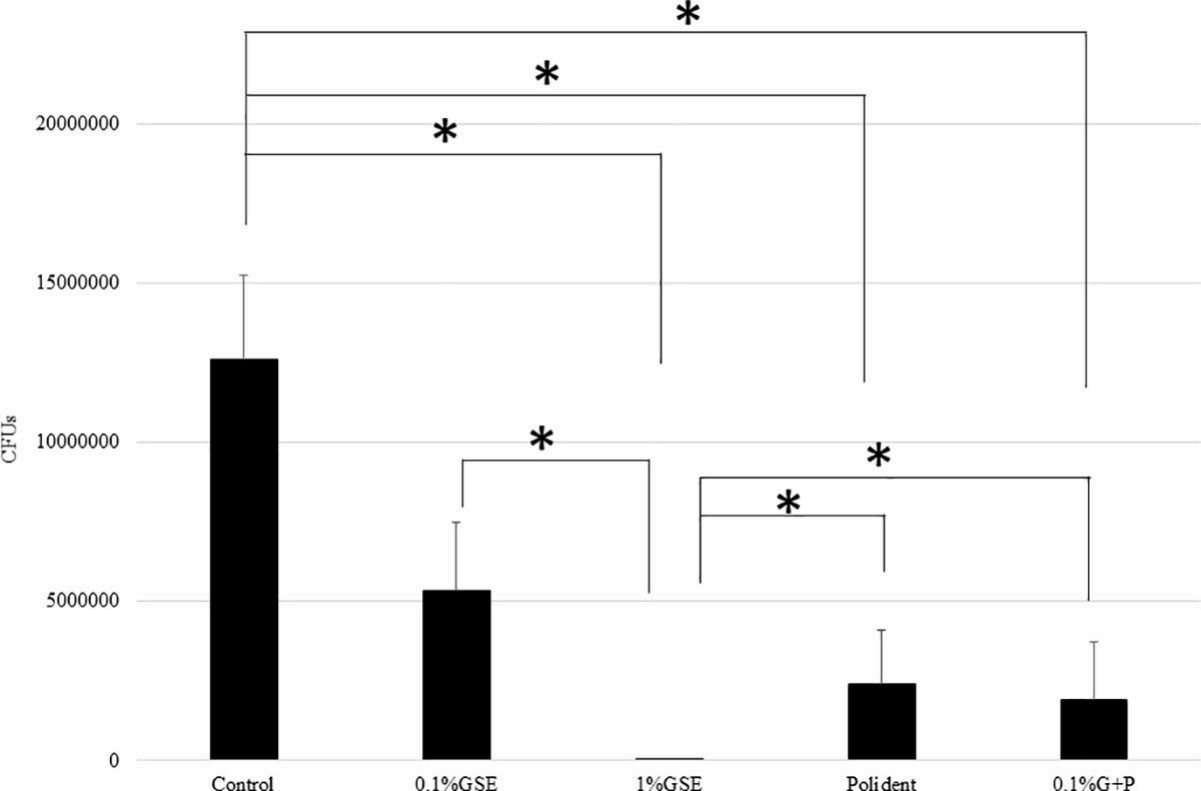
*C. albicans* biofilm quantification by CFU assay after treatment with each solution for 5 min. The black bars represent the mean colony count detected from specimens with *C*. *albicans* (n = 20 in each group). The asterisk (*) indicates a significant difference between groups (*p < 0*.*05*).

**Fig 2:**
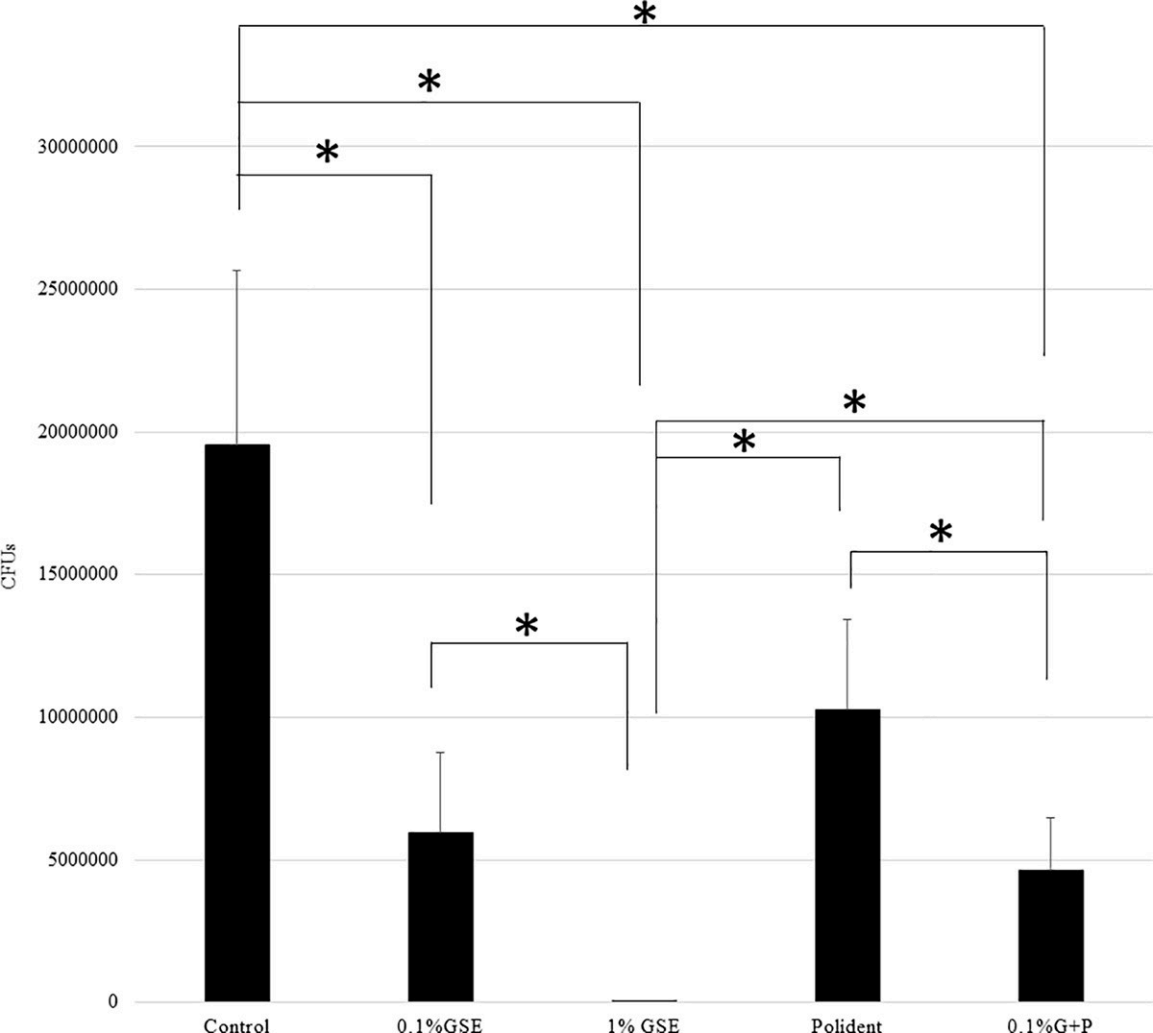
Quantification by CFU assay of *C*. *albicans* biofilm incubated for 24 h after treatment with each solution for 5 min. The black bars represent the mean colony count detected from specimens with *C*. *albicans* (n = 20 in each group). The asterisk (*) indicates a significant difference between groups (*p < 0*.*05*).

**Fig 3:**
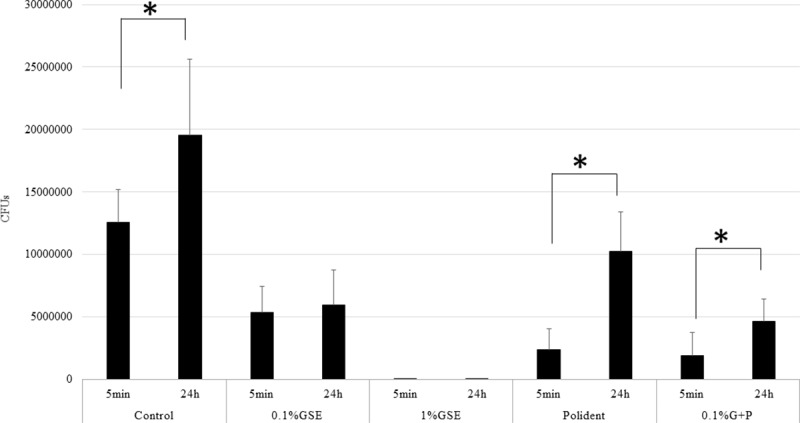
Comparison of *C*. *albicans* biofilm quantification by CFU assay after treatment for 5 min (n = 20 in each group) and after incubation for 24 h (n = 20 in each group). The results of 5 min in the control, Polident, and 0.1% G+P groups exhibited significantly lower average level of biofilm compared with those incubated for 24 h. The asterisk (*) indicates a significant difference between groups (*p < 0*.*05)*.

The CFU assays showed significantly lower quantities of biofilm on the discs from the 1% GSE, Polident, and 0.1% G+P groups than the control group. That is, the colony formation was inhibited by 57%, 98%, 80%, and 85% after treatment with 0.1% GSE, 1% GSE, Poldent and 0.1% G+P solutions, respectively. Among the cleansing solution groups, 1% GSE almost completely diminished the biofilm formed on disc surfaces compared with 0.1% GSE, Polident, and 0.1% G+P, among which no significant differences were noted (Fig 1). Another CFU assay to examine the persistent effect of each solution showed significantly lower quantities of biofilm on the discs from the 0.1% GSE, 1% GSE, and 0.1% G+P groups than the control group. That is, the colony formation was inhibited by 70%, 100%, 47%, and 76% after treatment with 0.1% GSE, 1% GSE, Poldent and 0.1% G+P solutions, respectively. Among the cleansing solution groups, the discs treated with the 1% GSE were significantly lower in biofilm content than those treated with 0.1% GSE, Polident, and 0.1% G+P. Interestingly, the addition of 0.1% GSE to Polident (0.1% G+P group) significantly inhibited biofilm formation compared with Polident alone. There were no significant differences among the 0.1% GSE, Polident, and 0.1% G+P groups (Fig 2). The comparison among the CFU assays for the discs treated with each solution followed by another 24-h incubation clearly revealed that treatment with 0.1% GSE and 1% GSE maintained the effectiveness of inhibition against biofilm formation even 24 h after a 5-min treatment. Importantly, the treatment with 1% GSE for 5 min almost eliminated the biofilm formed by *C*. *albicans* on the resins and inhibited the biofilm formation for at least 24 h after the initial treatment. Interestingly, the addition of 0.1% GSE to Polident (0.1% G+P) showed a more persistent effectiveness of inhibition of biofilm formation (Fig 3).

**Fig 4:**
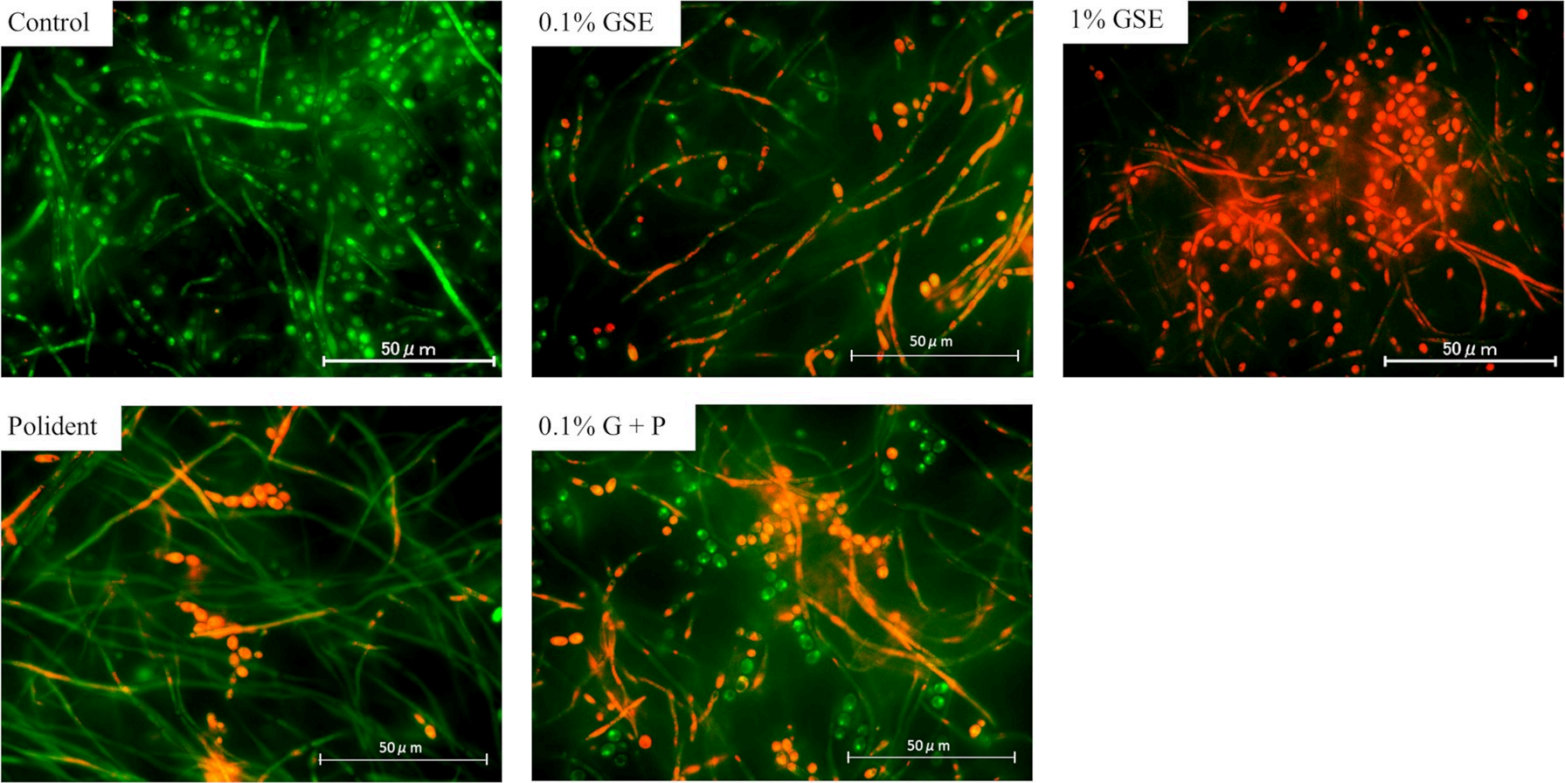
Biofilm on denture-base resin disc surface viewed using fluorescence microscopy to obtain a representative sample from each group. Green fluorescence showed living cells, and red fluorescence showed dead cells. The vast majority of fungus in the control groups appeared green; whereas, 1% GSE induced high mortality in the two morphogenetic forms, yeast and hyphae (increased red color). In the 0.1% GSE groups, dead cells were mainly observed in hyphal forms. The Polident groups showed cell death in yeast forms, but the vast majority of hyphal forms were alive (green). The 0.1% G+P groups showed many dead cells of the yeast and hyphal form.

Fluorescence microscopy, in which green fluorescence indicated living cells and red fluorescence indicated dead cells, revealed the viability of *C*. *albicans* cells inside the biofilms. Regardless of morphology (either yeasts or hyphae), the majority of fungus appeared green in the control group; whereas, in the 1% GSE group, almost every fungus appeared red in both yeasts and hyphae. In the other three groups (0.1% GSE, Polident, and 0.1% G+P), a mixture of red and green fluorescence with a different tendency among groups was observed. In detail, in the 0.1% GSE group, dead cells were observed in both yeast and hyphal forms; whereas, in the Polident group, cell death was noted predominantly in hyphal forms rather than yeast forms. In the 0.1% G+P group, more dead cells, regardless of the morphology, were observed (Fig 4).

**Fig 5:**
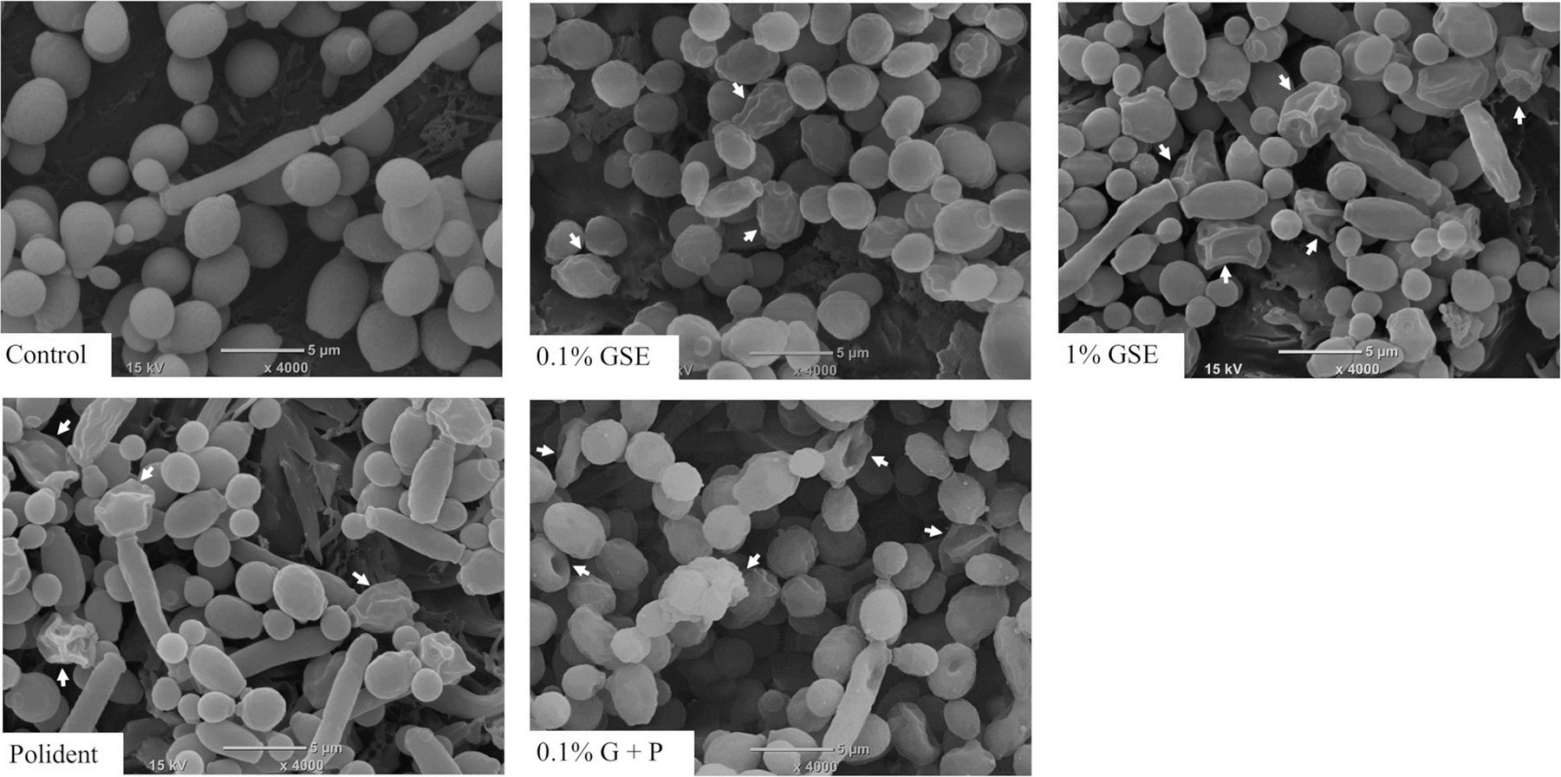
SEM images of the yeast forms inside *C*. *albicans* biofilms formed on representative denture-base resin disc surfaces from each group. White arrows indicate cell body shrinkage and deep craters on cell surfaces, which are not seen in the control.

**Fig 6:**
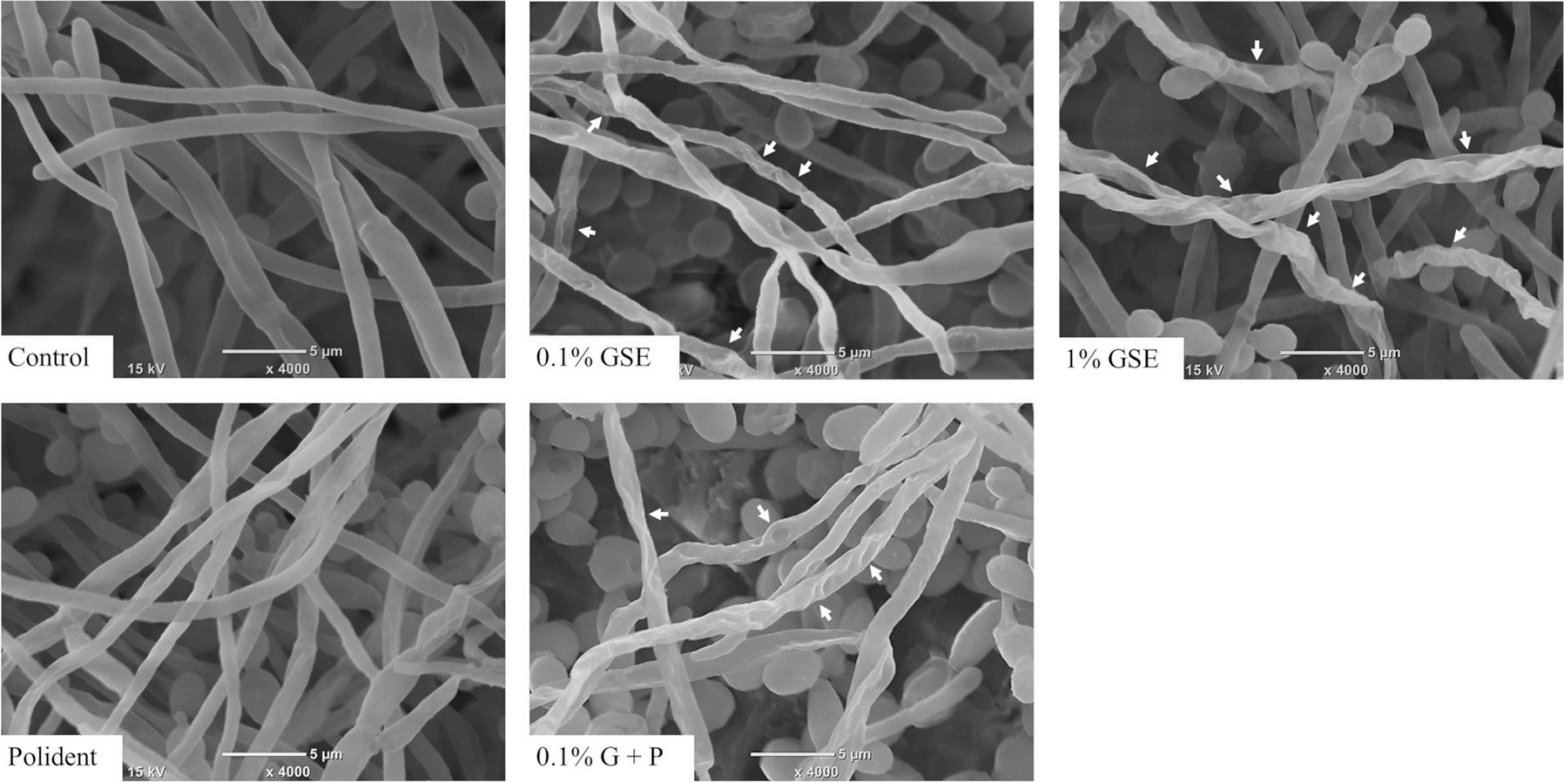
SEM images of the hyphae forms inside *C*. *albicans* biofilms formed on representative denture-base resin disc surfaces from each group. White arrows indicate shrinkage and damage on cell surfaces, which were not seen in the control and Polident.

The SEM images of *C*. *albicans* treated with each cleansing solution showed a variety of cell damage. Fig 5 shows the morphological changes of yeast forms of *C*. *albicans*, where wrinkles, shrinkage, and some deep craters were noted on the cell surfaces in all cleansing groups. In contrast, the evident signs of cell damages such as wrinkles and shrinkage on the surfaces of hyphae were observed only in the 0.1% GSE, 1% GSE, and 0.1%G+P groups. There was no notable difference in the hyphal surfaces between the Polident and control group (Fig 6).

Table 1 shows the mean values and standard deviation in Ra, VH, FS, and FM of the specimens after immersion in each solution. The values of Ra (n = 5), VH (n = 5), FS (n = 5), and FM (n = 5) were 0.28 to 0.39 μm, 21.25 to 22.51 Hv, 89.93 to 102.71 MPa, and 2.90 to 3.15 GPa, respectively, with no significant differences among the groups.

**Table 1:** Mean (SD) values of surface roughness (Ra), Vickers hardness (VH), flexural strength (FS), and flexural modulus (FM).

	Ra (μm)	VH (Hv)	FS (Mpa)	FM (Gpa)
Control	0.33 (0.13)	21.25 (1.37)	101.98 (7.00)	3.15 (0.02)
0.1% GSE	0.33 (0.16)	21.75 (0.93)	102.71 (5.07)	3.12 (0.08)
1% GSE	0.39 (0.17)	21.42 (1.82)	89.93 (7.42)	2.90 (0.27)
Polident	0.37 (0.10)	22.51 (3.02)	99.24 (7.07)	3.05 (0.14)
0.1% G+P	0.28 (0.16)	21.31 (0.69)	94.80 (7.13)	2.90 (0.26)

There were no significant differences among the groups in each analysis. (Ra: n = 5, VH: n = 5, FS and FM: n = 5, *p > 0*.*05*).

## Discussion

*C*. *albicans* biofilms recovered from discs treated with 0.1% GSE, 1% GSE, Polident, or 0.1% G+P for 5 min contained significantly much fewer vital *C*. *albicans* cells compared with those from the control group. Particularly, 1% GSE was proven to almost completely eliminate the vital cells from the biofilms. In addition, 0.1% GSE was nearly equally efficacious as Polident in the elimination of vital cells from biofilms. Microscopic images of 5-min treated *C*. *albicans* showed that this effect of GSE might be due to the cell death of *C*. *albicans*. Fluorescence microscopy observations showed that 1% GSE induced the death of *C*. *albicans* cells in both yeast and hyphal forms. Judging from the mechanism of the Live/Dead® FungaLight yeast viability kit, in which PI penetrates dead cells through the damaged cell wall/membrane and yields red fluorescence when it binds to DNA [26, 27], 1% GSE might have the potential to damage the *Candida* cell wall or membrane. Consistently, the SEM images of the 1% GSE-treated *C*. *albicans* showed shrinkage and surface damages in both yeasts and hyphae. The cell wall and cell membrane play important roles in cell viability, morphogenesis, response to environmental stressors, and pathogenesis [28]. Thus, these morphological changes are thought to reflect the breakdown of cell homeostasis, resulting in cell death. In contrast, in the 0.1% GSE, Polident, and 0.1% G+P groups, only a portion of yeast and hyphal forms of *C*. *albicans* were affected by each solution.

The antifungal mechanism of GSE has been studied using the yeast *Saccharomyces cerevisiae*. It has been reported that GSE induces apoptosis in the yeast cells by the destruction of the mitochondria [19], suggesting that the effect of GSE on *C*. *albicans* may be associated with cell apoptosis. Although a complete explanation for the antifungal mechanism of GSE on *C*. *albicans* is not feasible within the design of this study, the constituents that are responsible for the inhibitory effect of GSE on *C*. *albicans* may be inferred based on previous research. GSE contains flavonoids, tocopherol, ascorbic acid, citric acid, and a minute amount of other compounds [29]. Several studies have reported that flavonoids possess an antifungal property [13, 14, 30]. Flavonoids are a class of polyphenols, and they are subdivided into subclasses such as flavones, flavanols, isoflavones, and chalcones [31]. The mechanisms of action associated with the antifungal effects of flavonoids differ depending on these subclasses [16]. Flavones and isoflavones induce apoptosis via the inhibition of the efflux pump [32, 33]. Flavanols trigger cell wall damage [34], and chalcones induce cytoplasmic membrane disruption [35]. Other studies have also reported that phenolics induce alterations in cell membrane permeability because they compromise the cell membrane bilayers due to their lipophilic nature [36]. The change in membrane permeability causes an imbalance in the intracellular electrochemical gradients and subsequent depolarization, leading to the loss of membrane functions and cell death [37]. These studies suggest that several kinds of flavonoids in GSE could have additive/synergistic inhibitory effects on the cell wall and cell membrane of *C*. *albicans*. Further investigations are needed to clarify anti-fungal ingredients of GSE.

Next, we examined the persistent inhibitory effect of GSE on *C*. *albicans* biofilm development by using CFU assay of discs 24 h after the initial treatment with each solution. As a result, 1% GSE was proven to effectively inhibit the biofilm formation for at least 24 h after the initial treatment. The 0.1% GSE also exerted a persistent inhibitory effect. Interestingly, the addition of 0.1% GSE to Polident significantly inhibited biofilm formation compared with Polident alone. This effect of GSE on *C*. *albicans* may be assessed based on the fluorescence microscopic and SEM images in this study. The results of the fluorescence microscopy indicated that the Polident mainly induced the death of yeast, while the cleansing solutions contained at least 0.1% GSE induced the death of hyphae. In addition, SEM images of the yeast form treated with each solution showed wrinkles, shrinkage, and some deep craters on the surfaces, while the hyphae form treated with the solution containing at least 0.1% GSE showed wrinkles, shrinkage, and some damage on the surfaces. These microscopic images indicate that GSE inhibits the hyphae of *C*. *albicans* by an unknown mechanism. The hyphal growth plays an important role in biofilm maturation, so the inhibition of hyphal formation inhibits biofilm development [38]. Although hyphal development and growth are involved in several hyphae-specific genes, the inhibition of these genes induces the inhibition of hyphal development and biofilm formation [39, 40]. It has been reported that catechin, which is a subclass of flavonoids, inhibits hyphal formation by reducing mRNA expression levels of all hyphae-specific genes [39], suggesting that some types of flavonoids from GSE may persistently inhibit *C*. *albicans* growth by affecting some hyphae-specific genes. Therefore, the persistent inhibitory effect of GSE on *C*. *albicans* biofilm may be caused by the inhibition of both developed hyphae and new hyphal development.

In this study, the discs were treated with each solution for 5 min, and 1% GSE could almost completely inhibit *C*. *albicans* biofilm within the treatment time. This fact strongly suggests that treatment with 1% GSE for 5 min can exert an inhibitory effect even on the deepest layer; that is, the portion of the biofilm (consisting of multiple cell layers) closest to the surface of the discs. Although the reason underlying this phenomenon is not clear, one possibility is that some types of constituents contained in 1% GSE may induce the destruction of cell-cell/cell-matrix adhesion, which is important in forming the biofilm [41], at a higher speed than the other solutions. The continuous destruction of cell-cell/cell-matrix adhesion may be repeated as deep as the surface of discs, eventually resulting in the total inhibition of biofilms.

According to ISO 1567, the minimum FS of heat-polymerizing acrylic resin should be 65 MPa or more, and the minimum FM should be 2 GPa or more. In this study, FS was more than 89.93 MPa, and FM was more than 2.90 GPa after immersion for 7 days. Therefore, we complied with the requirement of ISO 1567 regarding both parameters. For VH and Ra, there were no significant changes before and after immersion. The VH test can represent the resistance of the surface of base materials against cleansing solutions, and surface alterations may be quantitatively measured with these tests [42]. It has been reported that surface degradation increases Ra [43]. Therefore, it is also evident that the immersion in each solution induces no surface deterioration.

Taken together, the present study clearly indicates the possibility that GSE may be an effective alternative measure for cleansing biofilms from denture surfaces. Although *C*. *albicans* is the most commonly isolated organism in denture plaque, denture plaque is composed of many types of bacteria and yeasts [44]. These microorganisms are considered to contribute to not only DS, oral malodor, caries, and periodontitis, but also to bacterial endocarditis, aspiration pneumonia, gastrointestinal infection, and chronic obstructive pulmonary disease [45]. Further investigations are needed to verify the inhibitory effect of GSE on the other microorganisms contained in denture plaque.

## Conclusion

The results of the present study strongly suggest that GSE can be very useful for the disinfection of denture surfaces. Our data also suggest that a denture treated with GSE for 5 min has the potential to have a persistent inhibitory effect on the biofilm development of *C*. *albicans*. In addition, immersion in the solution containing GSE does not cause surface deterioration of denture-base resin. Therefore, GSE has great potential as a new component of denture cleanser.

## Supporting information

S1 File CFU data overview. The data shows viable *C*. *albicans* cell numbers on the discs after treatment with each solution for 5 min.
(PDF)

S2 File CFU data overview. The data shows viable *C*. *albicans* cell numbers on the discs 24 h after the initial treatment with each solution.
(PDF)
