# Supplementary material for: Grapefruit seed extract effectively inhibits the Candida albicans biofilms development on polymethyl methacrylate denture-base resin
Source: PLoS One. 2019 May 28;14(5):e0217496. doi: 10.1371/journal.pone.0217496 (PMC6538181; doi:10.1371/journal.pone.0217496)
Supplement: S1 File — The data shows viable C. albicans cell numbers on the discs after treatment with each solution for 5 min. (PDF) [file pone.0217496.s001.pdf]

	control	GSE0.1%	GSE1%	polident	GSE+polident
1	13600000	4000000	0	4410000	80000
2	14600000	3100000	0	2570000	210000
3	12700000	6800000	0	3730000	410000
4	14800000	5400000	0	5420000	1660000
5	10800000	1600000	0	2880000	500000
6	15500000	1400000	0	3380000	520000
7	8100000	5200000	0	3240000	1270000
8	11800000	4100000	4	2700000	1280000
9	12200000	3100000	340	2670000	30000
10	8200000	4800000	1010	4240000	50000
11	14300000	8360000	250	790000	130000
12	15800000	4480000	2390	260000	1790000
13	18300000	7740000	1290	1500000	1210000
14	9900000	4480000	0	2060000	2740000
15	12300000	8100000	0	2370000	3850000
16	10000000	7720000	0	10000	3110000
17	14600000	5460000	230	0	5100000
18	11500000	8080000	180	160000	4200000
19	12500000	6460000	170	860000	4740000
20	10400000	6780000	140	4950000	5240000
average	12595000	5358000	300.2	2410000	1906000
