# Supplementary material for: Grapefruit seed extract effectively inhibits the Candida albicans biofilms development on polymethyl methacrylate denture-base resin
Source: PLoS One. 2019 May 28;14(5):e0217496. doi: 10.1371/journal.pone.0217496 (PMC6538181; doi:10.1371/journal.pone.0217496)
Supplement: S2 File — The data shows viable C. albicans cell numbers on the discs 24 h after the initial treatment with each solution. (PDF) [file pone.0217496.s002.pdf]

	control	GSE0.1%	GSE1%	polident	G+P
1	19400000	8340000	0	8700000	4800000
2	13000000	5250000	0	6700000	5900000
3	11600000	11960000	0	7200000	3600000
4	19600000	3000000	0	6800000	2800000
5	11100000	6000000	0	9200000	2100000
6	11000000	8400000	0	15800000	4700000
7	14400000	5600000	0	11900000	4900000
8	23100000	12300000	0	12600000	3200000
9	32100000	3050000	0	11400000	1900000
10	19600000	6700000	630	14000000	2800000
11	21400000	4400000	10	4100000	7000000
12	20400000	3300000	60	10300000	5400000
13	13400000	4900000	40	8500000	4800000
14	19400000	7100000	0	5400000	9000000
15	14200000	3100000	0	10100000	7000000
16	27500000	5100000	0	13700000	4000000
17	25500000	3300000	0	12000000	6000000
18	24200000	3400000	0	10900000	4100000
19	24600000	8500000	0	11900000	2800000
20	25800000	5500000	0	14300000	6000000
average	19565000	5960000	37	10275000	4640000
